# Robust closed-loop control of spike-and-wave discharges in a thalamocortical computational model of absence epilepsy

**DOI:** 10.1038/s41598-019-45639-5

**Published:** 2019-06-24

**Authors:** Yafang Ge, Yuzhen Cao, Guosheng Yi, Chunxiao Han, Yingmei Qin, Jiang Wang, Yanqiu Che

**Affiliations:** 1 School of Precision Instruments and Optoelectronics Engineering, Tianjin University, Tianjin, 300072 P. R. China; 2 School of Electrical and Information Engineering, Tianjin University, Tianjin, 300072 P. R. China; 3 Tianjin Key Laboratory of Information Sensing & Intelligent Control, School of Automation and Electrical Engineering, Tianjin University of Technology and Education, Tianjin, 300222 P. R. China; 4 Department of Neurosurgery, Penn State College of Medicine, Hershey, PA 17033 USA; 5 Center for Neural Engineering, Penn State, University Park, PA 16802 USA

**Keywords:** Dynamical systems, Epilepsy

## Abstract

In this paper, we investigate the abatement of spike-and-wave discharges in a thalamocortical model using a closed-loop brain stimulation method. We first explore the complex states and various transitions in the thalamocortical computational model of absence epilepsy by using bifurcation analysis. We demonstrate that the Hopf and double cycle bifurcations are the key dynamical mechanisms of the experimental observed bidirectional communications during absence seizures through top-down cortical excitation and thalamic feedforward inhibition. Then, we formulate the abatement of epileptic seizures to a closed-loop tracking control problem. Finally, we propose a neural network based sliding mode feedback control system to drive the dynamics of pathological cortical area to track the desired normal background activities. The control system is robust to uncertainties and disturbances, and its stability is guaranteed by Lyapunov stability theorem. Our results suggest that the seizure abatement can be modeled as a tracking control problem and solved by a robust closed-loop control method, which provides a promising brain stimulation strategy.

## Introduction

Epilepsy, a common chronic neurological disorder characterized by recurrent seizures, affects about 1% of people in the world^1^. Childhood absence epilepsy (CAE) is a form of generalized epilepsy commonly observed in children with transient impairment of consciousness and brief interruption of ongoing activities^2,3^. The most significant clinical manifestation of AE is the periodic 2.5–4 Hz spike-and-wave discharges (SWDs) in electroencephalography (EEG) in a bilateral, synchronous, and symmetric pattern^4,5^. Despite important progress on basic mechanisms of SWDs from experimental and clinical observations over the past few decades^6^, the underlying mechanisms responsible for the spontaneous transitions between normal ongoing activity and SWDs have not been fully understood. On the other hand, elimination of seizures as early as possible is critical for children to optimize their cognitive development and improve their behavior and quality of life^7^. As opposed to focal epilepsies, neurosurgical resection of epileptic foci area is generally not an option in patients with generalized epilepsies, while available anti-epileptic drug treatments often cause serious side-effects due to the chronic nature of the disorder^8^. Therefore, alternative seizure control methods are in great demand.

Computational models at different scales play a major integrative role in exploring the mechanisms underlying the initiation, evolution and abatement of epileptic seizures^9–12^. EEG recordings result from macroscopic ensemble dynamics of electrical activities over large scales of neural networks. Hence population models (neural mass models (NMMs) and neural field models (NFMs)), describing the average activity of interconnected subpopulations of principal neurons and interneurons, have been established from the 1950s to 1970s^13–17^. Over the last decades, a series of epilepsy and seizure models have been developed based on these seminal works^18–24^. Since many experimental and clinical recordings have shown that the subcortical thalamus is highly involved in the generation and evolution of generalized epilepsies^25–27^, thalamocortical computational models of epilepsy have been subsequently developed^19,22,24,28^. By extending the lumped model of alpha rhythm generation^15^, Suffczynski *et al*. proposed a network model consisting of mutually interconnected excitatory pyramidal cell population (PY) and inhibitory interneuron population (IN) in the cortex and the thalamocortical relay cells (TC) and reticular nucleus (RE) in the thalamus^19^. This model not only successfully reproduced the spontaneous occurring 11-7 Hz SWDs during the ongoing activity observed in AE rats^27^, but also provided useful insights into the interpretation of physiological mechanisms in AE. Motivated by this study and following Amari’s approach^17^, Taylor *et al*. presented a cortico-thalamic model with similar structure at the macroscopic level to account for SWDs dynamics of the cerebral cortex in AE humans with the aim to develop seizure abatement techniques^24^.

Epileptic seizures can be viewed from the perspective of dynamical diseases^29,30^. That is, the onset of a seizure is thought to correspond to the appearance of sustained high-amplitude nonlinear oscillations, suggesting a bifurcation from a steady state to a limit cycle or a chaotic attractor^11,19,22^. Specifically, the dynamics of SWDs can be understood by a bistable neuronal network, where the seizure state coexists with the background state^19,24,31^. The macroscopic models^19,24,31^ allow to examine dynamical properties of the system and different mechanisms for seizure generation. Bifurcation analyses of thalamocortical models revealed that Hopf bifurcations play a dominant role in governing the transitions from ongoing background state to SWDs or tonic-clonic seizures^19,22^. Recent experimental results in behaving animals have further demonstrated that paroxysmal oscillations of absence seizures are driven by top-down PY-to-TC excitation and framed by feedforward RE-to-TC inhibition^32^. However, how the bidirectional cortico-thalamic communications shape the seizure dynamics and its transitions have not been fully examined from a dynamical point of view.

For patients with drug-resistant epilepsy like AE, brain stimulation has been studied as a potential treatment option^33–36^. Most deep brain stimulation (DBS) protocols utilize open-loop control by continuously delivering high-frequency electrical pulses^37,38^. DBS has also been tested in computational models on controlling absence seizures and effective control could be obtained with properly targeted brain regions and fine-tuned stimulation parameters^39,40^. Compared to the open-loop methods, closed-loop brain stimulation, which could eliminate seizures without inducing side effects of continuous stimulation, has become a promising alternative^36,41–46^ in clinical trials^47,48^. Closed-loop transcranial electrical stimulation (TES)^36,41,42^, DBS^43,44^ and optogenetic stimulation^45,46^ can dramatically reduce SWDs in rodent models of generalized epilepsy. The clinically-utilized RNS System (*NeuroPace*, *Inc*. *USA*) shows at least comparable effectiveness with the traditional continuous DBS^47,48^. These closed-loop seizure control systems consisting of on-line seizure detection and real-time stimulation works as a responsive neurostimulator. That is, brain stimulation with pre-defined parameter settings is triggered when seizure events are detected. The underlying idea is to take advantages of the dynamical feature of multi-stability of neural system during seizures, with the hope of perturbing the brain state into the basin of attraction of normal background activities. For the SWDs in a bistable setting, it is possible to annihilate seizures by an appropriate stimulus delivered at a specific phase of the abnormal oscillations^11,19,24^. However, the effects of pre-defined stimulations on abating noise-induced SW seizures are highly dependent on several factors, such as the stimulation patterns, stimulation parameters and the timing of the stimulations^11,24,36,41–46,49^, which suggests that a robust closed-loop control approach with adaptive stimulation is necessary.

In this paper, we use the well developed thalamocortical neural mass model^24^ to investigate the mechanisms and control of absence seizures. Specifically, we first examine the underlying dynamical mechanisms of bidirectional cortico-thalamic effects on absence seizures using bifurcation analysis. Then we propose to formulate seizure elimination as a tracking control problem and use a closed-loop control framework. Considering the high nonlinearity, unmodeled dynamics and ubiquitous disturbances in the neural system, we design a hybrid control consisting of feedback control, radial basis function neural networks (RBFNN)^50^ and sliding mode control (SMC)^51^. With this control method, we can guarantee robust seizure control in presence of noises and uncertain external disturbances. The results presented in this paper can be used to construct an on-line closed-loop brain stimulation system for seizure abatements.

## Methods

### Thalamocortical model

Neural mass models can well characterize the macroscopic dynamics of neuronal networks and provide a potential way to illustrate the mechanisms underlying absence seizures. In this paper, we use a macroscopic model of thalamocortical system consisting essentially of the synaptic circuitry of thalamus and cortex to investigate the effects of stimulation on SWDs^24^.

**Figure 1:**
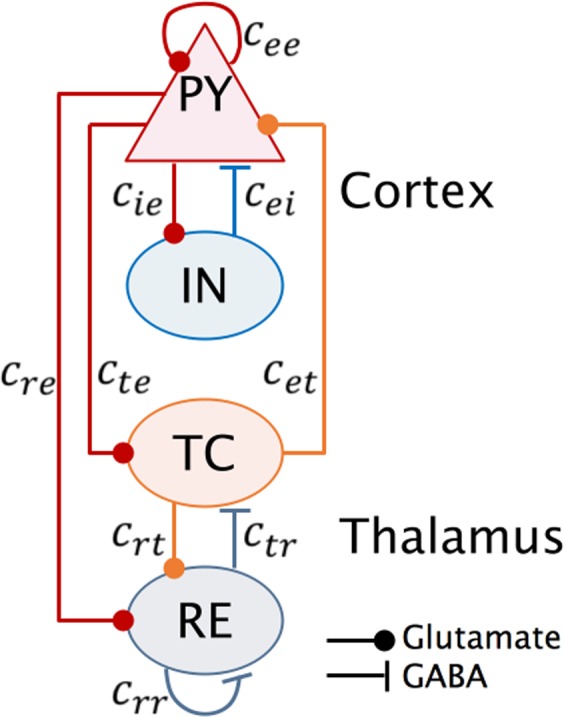
Schematic diagram of the neural field model of thalamocortical system used in this paper. It is composed of the cortical PY-IN subnetwork and the subcortical RE-TC subsystem. Lines with solid dots indicate the excitatory synaptic functions, lines with bars represent the inhibitory synaptic functions. PY: excitatory pyramidal neurons, IN: inhibitory interneurons, TC: thalamocortical relay cells, RE: thalamic reticular nucleus.

Figure 1 shows the connectivity scheme of the thalamocortical system^19,24^, where the cortical subsystem includes an excitatory pyramidal neuronal population (PY) and an inhibitory interneuronal population (IN), and the subcortical thalamus subsystem consists of the specific relay nucleus (TC) and thalamic reticular nucleus (RE)^19,24,49^. The interactions between different populations are represented by connectivity parameters *c*_*ee*,*ei*,*et*,*ie*,*te*,*tr*,*re*,*rt*,*rr*_. All populations are interconnected in agreement with experimentally known connections^26^.

The interactions within the thalamocortical system are described as the following differential equations:

1 $$\begin{array}{rcl}\frac{dPY}{dt} & = & \,{\tau }_{e}({h}_{e}-PY+{c}_{ee}f[PY]-{c}_{ei}f[IN]+{c}_{et}f[TC])\\ \frac{dIN}{dt} & = & \,{\tau }_{i}({h}_{i}-IN+{c}_{ie}f[PY])\\ \frac{dTC}{dt} & = & \,{\tau }_{t}({h}_{t}-TC+{c}_{te}f[PY]-{c}_{tr}s[RE])\\ \frac{dRE}{dt} & = & \,{\tau }_{r}({h}_{r}-RE+{c}_{re}f[PY]+{c}_{rt}s[TC]-{c}_{rr}s[RE])\end{array}$$

where *PY*, *IN*, *TC* and *RE* are the state variables, representing the fractional firing activity in each neuronal population. *h*_*e*,*i*,*t*,*r*_ are input parameters, *τ*_*e*,*i*,*t*,*r*_ are time scale constants mediated by different excitatory and inhibitory neuro-transmitters. *f* [*x*] = 1/(1 + *ε*^−*x*^) is the sigmoid transition function describing the cortical dynamics, and *s*[*x*] = *ax* + *b* is the linear activation function describing the thalamic subsystem. *c*_*ee*,*ei*,*et*,*ie*,*te*,*tr*,*re*,*rt*,*rr*_ are the connectivity strengths between different neuronal populations. The parameter values used in this paper are given in Table 1^24,49^.

**Table 1:** The parameter values used in this paper.

Parameter	Interpretation	Value
*h*_*e*_	*PY* input	−0.35
*h*_*i*_	*IN* input	−3.4
*h*_*t*_	*TC* input	−2
*h*_*r*_	*RE* input	−5
*τ*_*e*_	*PY* timescale	26
*τ*_*i*_	*IN* timescale	32.5
*τ*_*t*_	*TC* timescale	2.6
*τ*_*r*_	*RE* timescale	2.6
*c*_*ee*_	*PY* → *PY* connectivity strength	1.8
*c*_*ei*_	*IN* → *PY* connectivity strength	1.5
*c*_*et*_	*TC* → *PY* connectivity strength	1
*c*_*ie*_	*PY* → *IN* connectivity strength	4
*c*_*te*_	*PY* → *TC* connectivity strength	3, varied
*c*_*tr*_	*RE* → *TC* connectivity strength	0.6, varied
*c*_*re*_	*PY* → *RE* connectivity strength	3
*c*_*rt*_	*TC* → *RE* connectivity strength	10.5
*c*_*rr*_	*RE* → *RE* connectivity strength	0.2
*ε*	Sigmoid steepness	2 · 10^5^
*a*	Linear intersection steepness	2.8
*b*	Linear intersection offset	0.5

### Simulation methods

The numerical calculations were conducted in the MATLAB (*Math Works*, *USA*) simulation environment, where a standard fourth order Runge-Kutta method was used to solve differential equations, and the integration step was fixed at 1 ms. The cortical macroscopic dynamics were described by the simulated EEG signal *y*, which was calculated as the combination of excitatory and inhibitory population in cortex *y* = *c*_1_ · *PY* + *c*_2_ · *IN* with *c*_1_ > *c*_2_ > 0 and *c*_1_ + *c*_2_ = 1 the scaling factors. Although pyramidal cells have a greater open dipolar field influence than interneurons to the surface EEG^52,53^, a general demonstration of the effects of *c*_1_ and *c*_2_ given in Supplementary Fig. S1 shows that, the shape of the simulated EEG *y*(*t*) in different cases is almost consistent. For comparison with previous results^24,49^, we took *c*_1_ = *c*_2_ = 0.5 in the following simulations. The dominant frequency of the simulated EEG signals was calculated using Fast Fourier Transform (FFT). The complex state transition mechanisms were explored by using bifurcation analysis, where the bifurcation diagrams were calculated in Xppaut (http://www.math.pitt.edu/bard/xpp/xpp.html).

## Results

### Complex dynamics of epileptic seizures

In order to demonstrate the ictal bidirectional cortico-thalamic communications during absence seizures observed in recent experimental results^32,54,55^, in this subsection, we investigate the combined effects of cortical excitation and thalamic inhibition on the onset and elimination of SWDs. Therefore, the parameters *c*_*te*_ and *c*_*tr*_ in Eq. (1) are taken as the key parameters to reveal how they affect the transition dynamics of the model.

**Figure 2:**
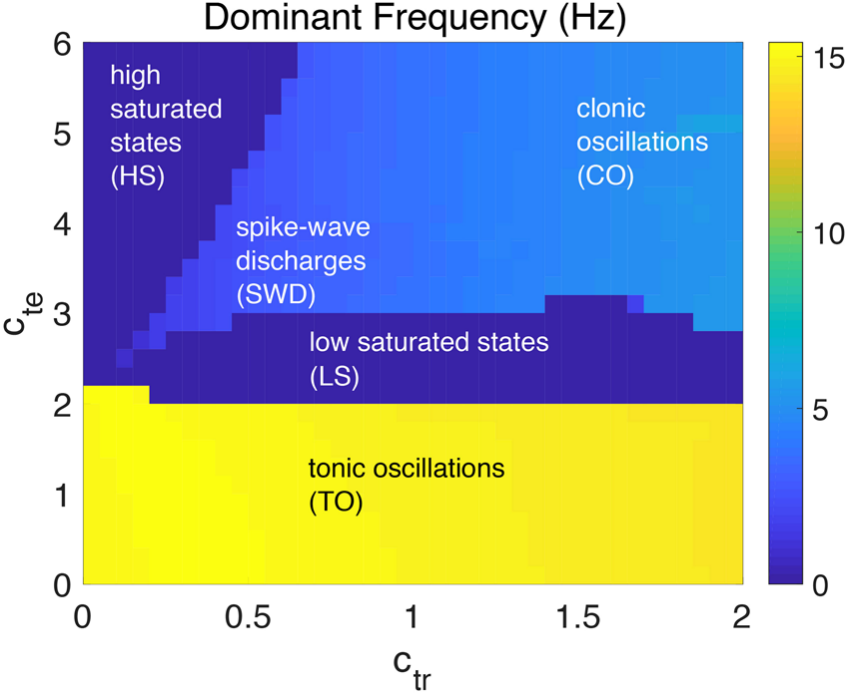
Distribution of different dominant frequency and corresponding states of the output in the parameter space (*c*_*tr*_, *c*_*te*_). There are several different types of states, including high saturated states (HS), spike-and-wave discharges (SWD), clonic oscillations (CO), low saturated states (LS) and tonic oscillations (TO).

**Figure 3:**
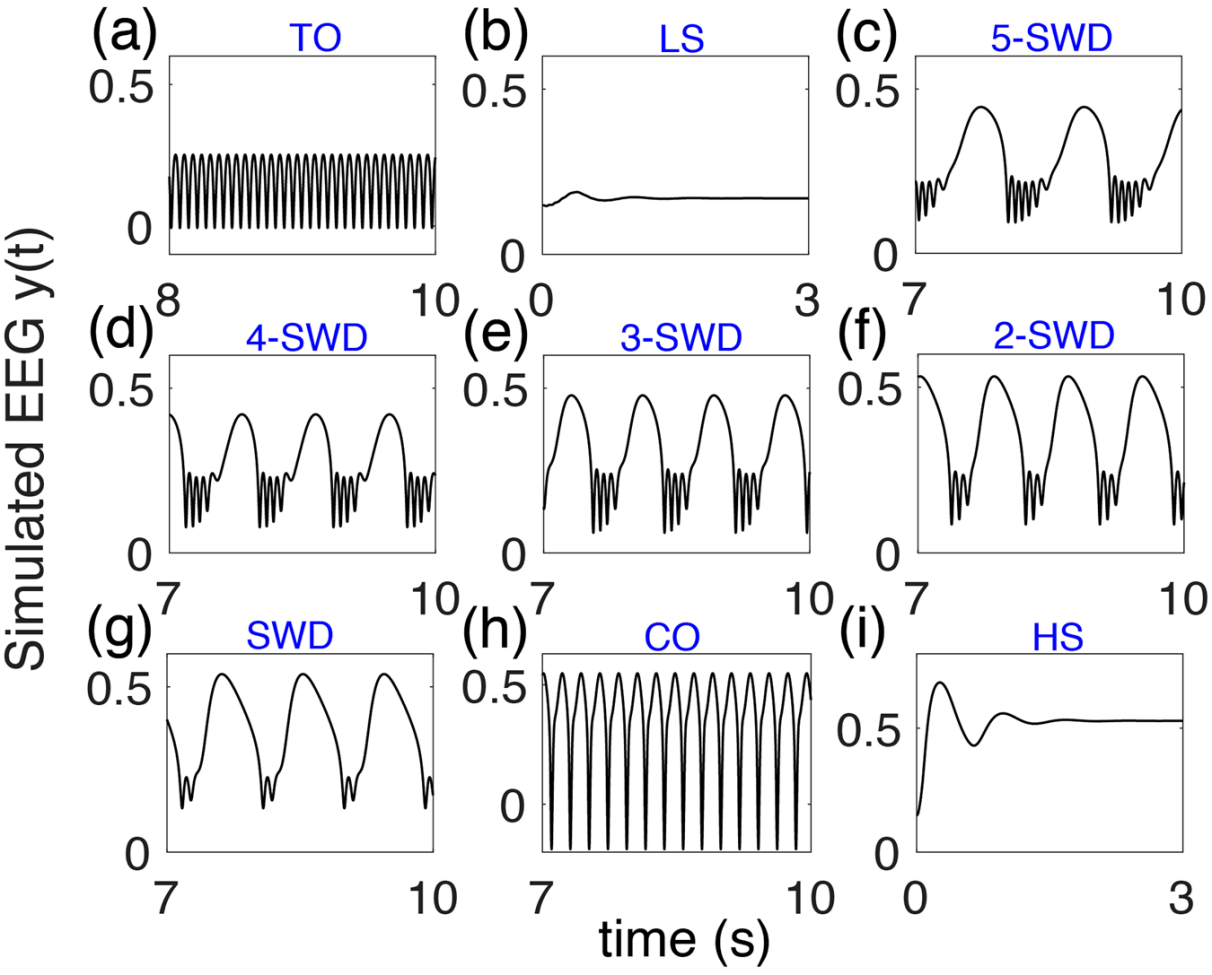
Different types of states with certain parameters in the thalamocortical model. TO: tonic oscillations, LS: low saturated state (normal background activity), m-SWD: m-spikes and wave discharge activity (here m can be 1, 2, …, 5), CO: clonic oscillations, HS: high saturated state.

Given certain parameter values and proper initial states, the model may inherently produce several qualitatively different behaviors without a stimulus, including spontaneous SWDs. Figure 2 shows the overall distribution of dominant frequencies and corresponding states of the thalamocortical model in the parameter space (*c*_*tr*_, *c*_*te*_). Figure 3 gives typical waveforms of the simulated EEG. With changes of the parameter values, the model can generate various states and induce several different types of transitions between them. We can see clear boundaries among different regions of states and dominant frequencies. In particular, there are five different states: tonic oscillations (TO), low saturated steady state (LS), high saturated steady state (HS), spike-and-wave discharges (SWD), and clonic oscillations (CO). Roughly speaking, when the value of parameter ${c}_{{te}}$ is small enough (*c*_*te*_ < 2), no matter how the value of parameter *c*_*tr*_ changes, the simulated EEG is basically in pathological tonic oscillations with a very high frequency and relatively low amplitude (Fig. 3(a), *c*_*tr*_ = 0.15 and *c*_*te*_ = 1). This may correspond to the interaction conditions within the cortex and thalamus that the excitatory synaptic connection of the loop from cortex to the TC in thalamus plays the role and the thalamus can not successfully receive information from the cortex when *c*_*te*_ is too weak. With slightly bigger *c*_*te*_, LS states dominate in a wide range of *c*_*tr*_, which correspond to normal background activities (Fig. 3(b), *c*_*tr*_ = 0.15 and *c*_*te*_ = 2.3). In the region with big *c*_*te*_ and small *c*_*tr*_, pathological HS states would occur (Fig. 3(h), *c*_*tr*_ = 0.15 and *c*_*te*_ = 3). With medium values of *c*_*te*_ and *c*_*tr*_, the typical SWD and multi-spike-and-wave discharges ($m$-SWD) can be observed as that in absence epilepsy^56^ (Fig. 3(c), 5-SWD, *c*_*tr*_ = 0.1 and *c*_*te*_ = 2.36; (d) 4-SWD, *c*_*tr*_ = 0.15 and *c*_*te*_ = 2.47; (e) 3-SWD, *c*_*tr*_ = 0.15 and *c*_*te*_ = 2.5; (f) 2-SWD, *c*_*tr*_ = 0.15 and *c*_*te*_ = 2.65; (g) SWD, *c*_*tr*_ = 0.15 and *c*_*te*_ = 2.17). For large values of *c*_*te*_ and *c*_*tr*_, CO are the dominant phenomena (Fig. 3(i), *c*_*tr*_ = 1.5 and *c*_*te*_ = 3.5).

**Figure 4:**
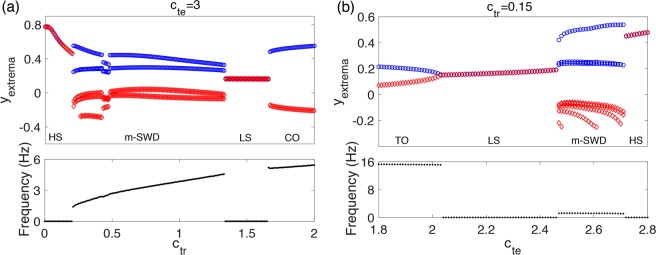
Bifurcation diagrams: the extrema of the simulated EEG ***y*** and the corresponding transitions of dominant frequency. (**a)** The dynamics transitions of the system over changes in *c*_*tr*_ with *c*_*te*_ = 3. **(b)** The dynamics transitions of the system over changes in *c*_*te*_ with *c*_*tr*_ = 0.15. The transitions of states include HS, *m*-SWD, LS, CO, and TO, where *m* can be 1, 2, …, 6.

To illustrate how the changes of synaptic coupling strengths affect the transitions of different states, as shown in Fig. 4, we calculate extreme values of simulated EEG (*y*_*extrema*_) in dominant states and corresponding frequencies as functions of one parameter *c*_*tr*_ (Fig. 4(a), *c*_*te*_ = 3) or *c*_*te*_ (Fig. 4(b), *c*_*tr*_ = 0.15). At a moderate strength of top-down excitation from PY to TC (*c*_*te*_ = 3), increase in connection strength of feedforward inhibition from RE to TC will induce state transitions from HS to 2.5–4 Hz *m*-SWD (typically observed in absence epilepsy), to normal background activities (LS) and even CO (typically observed in tonic-clonic epilepsy). On the other hand, with a very weak feedforward inhibition from RE to TC (*c*_*tr*_ = 0.15), a series of transitions from high-frequency TO to normal LS to low-frequency *m*-SWD, and to pathological HS can also be observed.

#### Bifurcation analysis

**Figure 5:**
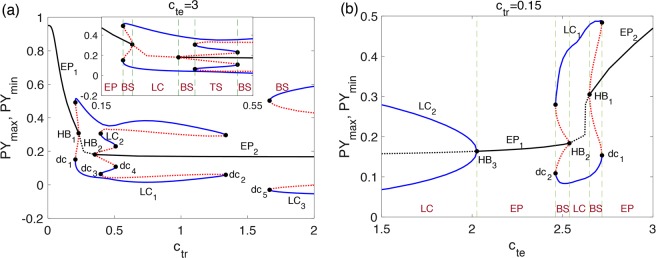
Bifurcation diagrams: the maximum and minimum of state *PY*. (**a**) The dynamics transitions of the system over changes in *c*_*tr*_ with *c*_*te*_ = 3. **(b)** The dynamics transitions of the system over changes in *c*_*te*_ with *c*_*tr*_ = 0.15. The transitions of states include HS, *m*-SWD, LS, CO, and TO, where *m* can be 1, 2, …, 5. BS is bi-stability, TS is tri-stability, HB is Hopf bifurcation and dc represents double cycle bifurcation.

To further explore the dynamical mechanisms underlying the above state transitions, we conducted bifurcation analysis. Figure 5 shows the one-parameter bifurcation diagrams corresponding to Fig. 4, where equilibrium points (EP) are corresponding to the steady states (HS and LS), while limit cycles (LC) are corresponding to the repetitive pathological states (SWD, TO, and CO). There exist several different multi-stable regions with varying *c*_*tr*_ or *c*_*te*_. BS, TS, HB and dc represent bi-stability, tri-stability, Hopf bifurcation and double cycle bifurcation, respectively. In Fig. 5(a) it is illustrated that the system transits within the monostable, bistable and triple stable states as *c*_*tr*_ changes. At HB_1_, the stability of equilibrium point EP_1_ changes from stable to unstable, and then an unstable limit cycle appears. The amplitude of the unstable LC becomes larger and finally coalesces with stable limit cycle (LC_1_) at bifurcation point dc_1_. Thus, in the range between dc_1_ and HB_1_, there exists bi-stability of EP and LC, corresponding to the coexistence of HS and SWD states. Similarly, the coexistence of LS and SWD states occurs in the range between HB_2_ and dc_3_ and between dc_4_ and dc_2_, while the coexistence of LS and CO states occurs when *c*_*tr*_ is beyond dc_5_. Note that in the range between dc_3_ and dc_4_, there exists tri-stability of one stable EP (EP_2_) and two stable LCs (LC_1_ and LC_2_), corresponding to coexistence of LS and two types of *m*-SWDs. In Fig. 5(b), the amplitude of stable limit cycle becomes smaller with the increase of *c*_*te*_, and finally disappears at bifurcation point HB_3_. Meanwhile, the stability of the equilibrium point changes from unstable to stable. Coexistence of LS and SWD (between HB_2_ and dc_2_), and HS and SWD (between HB_1_ and dc_1_) can also be observed in certain ranges of *c*_*te*_.

**Figure 6:**
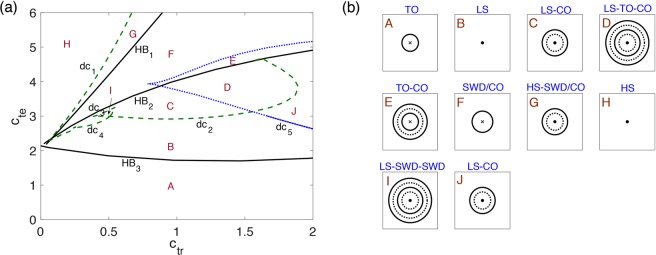
Two-parameter bifurcation diagrams in the *c*_*tr*_ − *c*_*te*_ space. (**a)** The bifurcation curves separate the parameter space into 10 qualitatively different regions (A–J). **(b)** Schematic phase portraits and corresponding states in different regions.

Figure 6 shows the two-parameter bifurcation diagram of the thalamocortical model in *c*_*tr*_ − *c*_*te*_ space. Hopf bifurcations (HB_1_, HB_2_, HB_3_) and double cycles (dc_1_, dc_2_, dc_3_, dc_4_ and dc_5_) are represented as solid and dashed lines, respectively. Therefore, the two-paramater space in Fig. 6(a) is partitioned into 10 qualitatively different regions (A–J) by these curves. Figure 6(b) gives a schematic phase portrait and corresponding states in each region. Among these regions, only in region B, the system behaves as normal background activities. The system states are very sensitive to system parameters and there are many pathways to pathological activities. For example, too weak excitation from PY to IN will induce high-frequency tonic oscillations (TO in region A), while too strong excitation from PY to IN may induce SWD (in regions C, F and G) observed in absence epilepsy or even tonic-clonic seizures (in regions C, D, F and G). In the bi-stable (C, E, G and J) or tri-stable (D and I) regions, the system behavior is very sensitive to the initial states or external disturbances.

#### Stimulation induced state transitions

Previous studies have shown that, a single-pulse stimulation can induce the onset and termination of SWDs in this model^24^. The results depend not only on the stimulus but also on the timing of the stimulus applied. Here, we further demonstrate the difficulties in using open-loop stimulation to eliminate SWD seizures.

**Figure 7:**
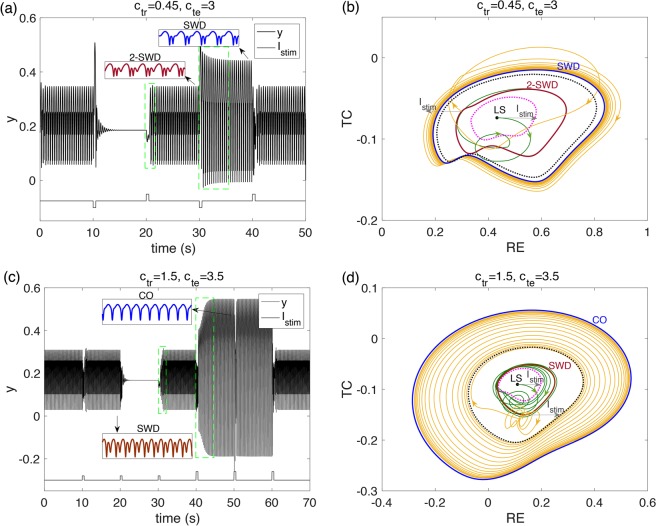
Single-pulse stimulation induced state transitions among pathological SWD or 2-SWD, normal background state and CO. (**a)** *c*_*tr*_ = 0.45, *c*_*te*_ = 3, time series of simulated EEG, **(b)** phase portrait on RE-TC plane corresponding to (**a**). **(c)** *c*_*tr*_ = 1.5, *c*_*te*_ = 3.5, time series of simulated EEG, **(d)** phase portrait on RE-TC plane corresponding to (**c**). The dotted circles in phase portraits (**b**) and (**d**) are the boundaries of different attractors.

As shown in Fig. 7(a,b), we chose a pair of parameters in region I (*c*_*tr*_ = 0.45, *c*_*te*_ = 3), where triple stability exists, that is, coexistence of LS, 2-SWD and SWD. Then we applied pulses stimulation on RE and observe the state transitions. The system initially exhibits 2-SWD state, and a properly chosen negative single pulse (applied at *t* = 10) successfully induced the system to normal background activities. However, a positive single pulse drove the system back to pathological 2-SWD. Moreover, when the same single pulse as the first one was applied to the system, it not only failed to abate the seizures, but even induced SWD with a larger amplitude. Figure 7(c,d) shows an another experiment. The parameter set was chosen in the region D (*c*_*tr*_ = 1.5, *c*_*te*_ = 3.5), where three stable states, LS, SWD and CO coexist. Although the second single pulse succeeded to abate the SWD, the first one failed and the third one induced the system back to SWD again, indicting the normal LS state is very sensitive to external disturbances. Single pulses with a bigger amplitude also couldn’t guarantee successful abatement of seizures. They may even cause more serious clonic oscillations (the fourth pulse).

Overall, the single-pulse applied on RE can change the oscillation dynamics among pathological SWD (2-SWD), LS or CO through the pathway RE-TC-Cortex, which can be attributed to the regulatory mechanism of the tri-stability in Fig. 7(b,d). The pulses stimulation can push the system state across the basin of attraction (dotted circles in phase portraits (b) and (d)). Although the single-pulses applied on RE can induce the transition from pathological SWDs to normal background resting states, the same stimulation may also induce transitions from normal activity to pathological SWDs. Moreover, the excitability, bi-stability or tri-stability of the system highly depend on initial parameter conditions and stimulation setting. Therefore, it is unreliable to use open-loop single-pulse stimulation to eliminate the pathological SWD during absence seizures, and we need to seek a more comprehensive close-loop control strategy.

### Seizure abatement using feedback control

Due to the complex dynamics of epilepsy system, the success of open-loop stimulation for seizure abatement is highly dependent of the stimulus itself, the system states and the stimulation timing^24,41,49^. For example, the previous proposed optimal pulse stimulation control is required to be applied at certain phase of the SWD oscillation, which is very difficult to detect. Here, we focus on a close-loop strategy.

#### Seizure abatement problem formulation

We first formulate the seizure abatement to a tracking control problem. We assume that there exists a cortical area exhibiting normal background activities. We use the measured normal EEG to represent the desired brain activity, and design a control system to force the cortical areas with pathological dynamics to behave similar dynamics of the measured normal EEG. Thus, the seizures can be abated.

In the thalamocortical model, the system under control can be described as a general differential equation:

2 $$\dot{{\bf{x}}}(t)={\bf{F}}({\bf{x}}(t))+{\bf{d}}(t)+{\bf{u}}(t)$$

where **x** = (*x*_1_, *x*_2_, *x*_3_, *x*_4_)^*T*^ = (*PY*, *IN*, *TC*, *RE*)^*T*^ is the system state, **F** = (*F*_1_, *F*_2_, *F*_3_, *F*_4_)^*T*^ is the mapping function as described in Eq. (1), **d** = [*d*_1_, *d*_2_, *d*_3_, *d*_4_]^*T*^ ∈ *R*^4^ is the disturbance and **u** = [*u*_1_, *u*_2_, *u*_3_, *u*_4_]^*T*^ is the controller. Here, we apply our control on PY and IN subpopulations, simulating external stimulations on the cortical area, that is *u*_1_ = *u*_2_ = *u* and *u*_3_ = *u*_4_ = 0.

The system output is defined as the simulated EEG, *y* = *c*_1_*x*_1_ + *c*_2_*x*_2_. The desired state *y*_*d*_ can be measured from a normal minicolumn. The goal is to control the system output *y* to track the desired state *y*_*d*_. We will design a neural network based sliding mode control, which guarantees stable tracking under uncertain dynamics and external disturbances.

#### Traditional sliding mode controller design

Sliding mode control (SMC) is characterized by a discontinuous control action that alters the dynamics of a nonlinear system by forcing the system to a set of predetermined sliding surfaces^51^. When reaching the sliding surface, the motion is independent of parameters and disturbances, resulting in a very robust system^57^.

We define the tracking error as *e* = *y* − *y*_*d*_, then its dynamics is

3 $$\begin{array}{rcl}\dot{e} & = & \,\dot{y}-{\dot{y}}_{d}\\  & = & \,{c}_{1}{\dot{x}}_{1}+{c}_{2}{\dot{x}}_{2}-{\dot{y}}_{d}\\  & = & \,{c}_{1}({F}_{1}({\bf{x}})+{d}_{1})+{c}_{2}({F}_{2}({\bf{x}})+{d}_{2})+u-{\dot{y}}_{d}\\  & = & \,g({\bf{x}})+d+u-{\dot{y}}_{d}\end{array}$$

where *g*(**x**) = *c*_1_*F*_1_(**x**) + *c*_2_*F*_2_(**x**) and *d* = *c*_1_*d*_1_ + *c*_2_*d*_2_.

Following the traditional sliding mode control design, we first choose the sliding surface as *s*(*e*, *t*) = *e*(*t*). As long as the system operates in the sliding mode, it satisfies the equations $s(t)=\dot{s}(t)=0$^51^, thus $e(t)=\dot{e}(t)=0$. Then we choose the reaching law as a constant-rate one, $\dot{s}=-\,\lambda s$, where parameter *λ* > 0 is determined such that the sliding condition is satisfied and the sliding mode motion occurs. Now the desired sliding mode control input is determined as

4 $$\begin{array}{l}{u}_{s}(t)=-\,\lambda s+{u}_{eq}\end{array}$$

where *u*_*eq*_ is equivalent control to deal with *g*(**x**), *d* and ${\dot{y}}_{d}$ in Eq. (3).

The *u*_*eq*_ can be selected as

5 $$\begin{array}{l}{u}_{eq1}=-\,\rho {\rm{sign}}(s)\end{array}$$

where

6 $$\begin{array}{l}{\rm{sign}}(s)=\{\begin{array}{ll}1, & {\rm{if}}\,s\ge 0\\ -\,1, & {\rm{if}}\,s < 0\end{array}\end{array}$$

Then the actual control is designed as

7 $$\begin{array}{l}{u}_{a1}(t)=-\,\lambda s+{u}_{eq1}=-\,\lambda s-\rho {\rm{sign}}(s)\end{array}$$

Consider the following candidate Lyapunov function

8 $$\begin{array}{l}V=\frac{1}{2}{s}^{2}\end{array}$$

The derivative of *V* is computed as

9 $$\dot{V}=s\dot{s}=s(g({\bf{x}})+d+u-{\dot{y}}_{d})$$

Let $u={u}_{a1}$, then we have

10 $$\begin{array}{rcl}\dot{V} & = & s(-\,\lambda s-\rho {\rm{sign}}(s)+g({\bf{x}})+d-{\dot{y}}_{d})\\  & = & -\lambda {s}^{2}-\rho |s|+s(g({\bf{x}})+d-{\dot{y}}_{d})\\  & \le  & -|s|(\lambda |s|+\rho -L)\end{array}$$

where *L* is the boundary of $g({\bf{x}})+d+{\dot{y}}_{d}$. Thus the stability condition $\dot{V}\le 0$ is always satisfied when *ρ* ≥ *L*.

#### RBFNN based controller design

Another method to design *u*_*eq*_ is to use a RBFNN model to approximate the unknown function *g*(*x*) and deal with the disturbance by properly choosing the robust adaptive update laws. A RBFNN is an artificial neural network with RBFs as the activation functions and a linear combination of these RBFs as the output. According to approximation theory, an unknown nonlinear smooth function *g*(**x**):**R**^*n*^ → **R** can be approximated by the RBFNN *g*_*nn*_(*x*) = ***θ***^*T*^***ϕ***(**x**)^58^, where the input vector *x* ∈ **Ω**_**z**_ ⊂ ***R***^*n*^ with **Ω**_**x**_ being a compact set, the weight vector ***θ*** ∈ **Ω**_***θ***_ ⊂ **R**^*m*^ with *m* being the NN node number, and basis function ***ϕ***(**x**) chosen as the commonly used Gaussian functions with fixed centers and widths. According to^50^, ***θ***^*T*^***ϕ***(**x**) with sufficiently large number of NN nodes can approximate any continuous function *f*(**x**) over the compact set **Ω**_**x**_ to arbitrary accuracy in form of

11 $$g({\bf{x}})={{\boldsymbol{\theta }}}^{\ast T}{\boldsymbol{\varphi }}({\bf{x}})+\varepsilon ,\,\,\forall x\in {{\boldsymbol{\Omega }}}_{x}$$

where θ^*^ is the ideal constant weight vector, and *ε* is the approximate error which is assumed to have an upper bound.

Typically, θ^*^ is chosen as the value that minimizes |*ε*| for all **x** ∈ **Ω**_**x**_, i.e.

12 $$\begin{array}{l}{{\boldsymbol{\theta }}}^{\ast }:={\rm{\arg }}\mathop{{\rm{\min }}}\limits_{{\boldsymbol{\theta }}\in {{\boldsymbol{\Omega }}}_{{\boldsymbol{\theta }}}}[\mathop{{\rm{\sup }}}\limits_{x\in {{\boldsymbol{\Omega }}}_{x}}|{{\boldsymbol{\theta }}}^{\ast T}{\boldsymbol{\varphi }}({\bf{x}})-g({\bf{x}})|]\end{array}$$

Since θ^*^ is generally unknown and needs to be estimated in controller design, let $\hat{{\boldsymbol{\theta }}}$ be the estimate of θ^*^, and denote $\tilde{{\boldsymbol{\theta }}}=\hat{{\boldsymbol{\theta }}}-{{\boldsymbol{\theta }}}^{\ast }$ as the weight estimate error vector.

**Assumption 1**. The ideal weight vector θ^*^ is bounded by an unknown positive value *θ*_*M*_ so that ||θ^*^|| ≤ *θ*_*M*_

We choose the following adaptive update laws for NN weights:

13 $$\dot{\hat{{\boldsymbol{\theta }}}}=-\,\gamma s{\boldsymbol{\varphi }}-{k}_{c}\gamma |s|\hat{{\boldsymbol{\theta }}}$$

where *k*_*c*_ is a given constant.

Sliding variable *s* will be used as a single input signal for establishing an RBFNN model to calculate the control law *u*_*eq*_(*t*)

14 $$\begin{array}{l}{u}_{eq2}=-\,{\hat{{\boldsymbol{\theta }}}}^{T}{\boldsymbol{\varphi }}(s)\end{array}$$

Then the actual control is designed as

15 $$\begin{array}{l}{u}_{a2}(t)=-\,\lambda s+{u}_{eq2}=-\,\lambda s-{\hat{{\boldsymbol{\theta }}}}^{T}{\boldsymbol{\varphi }}(s)\end{array}$$

Consider the following Lyapunov function candidate

16 $$\begin{array}{l}V=\frac{1}{2}{s}^{2}+\frac{1}{2\gamma }{\tilde{{\boldsymbol{\theta }}}}^{T}\tilde{{\boldsymbol{\theta }}}\end{array}$$

Differentiating Eq. (16) with respect to time and noting Eqs (13) and (15), we obtain

17 $$\begin{array}{rcl}\dot{V} & = & s\dot{s}+\frac{1}{\gamma }{\tilde{{\boldsymbol{\theta }}}}^{T}\dot{\hat{{\boldsymbol{\theta }}}}\\  & = & -\,\lambda {s}^{2}-s({\tilde{{\boldsymbol{\theta }}}}^{T}{\boldsymbol{\varphi }}+\omega )+\frac{1}{\gamma }{\tilde{{\boldsymbol{\theta }}}}^{T}(-\,\gamma s{\boldsymbol{\varphi }}-{k}_{c}\gamma |s|\hat{{\boldsymbol{\theta }}})\\  & = & -\,\lambda {s}^{2}-{k}_{c}|s|{\tilde{{\boldsymbol{\theta }}}}^{T}\hat{{\boldsymbol{\theta }}}+s\omega \end{array}$$

According to *Assumption 1*, we have

18 $${\tilde{{\boldsymbol{\theta }}}}^{T}\hat{{\boldsymbol{\theta }}}={\tilde{{\boldsymbol{\theta }}}}^{T}(\tilde{{\boldsymbol{\theta }}}+{{\boldsymbol{\theta }}}^{\ast })\ge \parallel \tilde{{\boldsymbol{\theta }}}{\parallel }^{2}-\parallel \tilde{{\boldsymbol{\theta }}}\parallel \parallel {{\boldsymbol{\theta }}}^{\ast }\parallel \ge \parallel \tilde{{\boldsymbol{\theta }}}{\parallel }^{2}-\parallel \tilde{{\boldsymbol{\theta }}}\parallel {\theta }_{M}$$

Applying the above inequality to Eq. (17), we have

19 $$\dot{V}\le -\,|s|[\lambda |s|+{k}_{c}(\parallel \tilde{{\boldsymbol{\theta }}}{\parallel }^{2}-\parallel \tilde{{\boldsymbol{\theta }}}\parallel {\theta }_{M})-{\omega }_{N}]$$

which is negative as long as the term in square bracket is positive.

Completing the square for the term inside the square bracket in Eq. (19) yields

20 $$\begin{array}{c}\lambda |s|+{k}_{c1}(\parallel \tilde{{\boldsymbol{\theta }}}{\parallel }^{2}-\parallel \tilde{{\boldsymbol{\theta }}}\parallel {\theta }_{M})-{\omega }_{N}\\ =\,{k}_{c}{(\parallel \tilde{{\boldsymbol{\theta }}}\parallel -{\theta }_{M}/2)}^{2}-{k}_{c}{{\boldsymbol{\theta }}}_{M}^{2}/4+\lambda |s|-{\omega }_{N}\end{array}$$

which is positive as long as

21 $$\begin{array}{l}|s| > ({k}_{c}{\theta }_{M}^{2}/4+{\omega }_{N})/\lambda \end{array}$$

or

22 $$\begin{array}{l}\parallel \tilde{{\boldsymbol{\theta }}}\parallel  > {\theta }_{M}/2+\sqrt{({k}_{c}{\theta }_{M}^{2}/4+{\omega }_{N})/{k}_{c}}\end{array}$$

Thus, $\dot{V}$ is negative outside a compact set. According to a standard Lyapunov theorem extension^59^, this demonstrates the uniformly ultimately bounded (UUB) of both ∥**e**∥ and $\parallel \tilde{{\boldsymbol{\theta }}}\parallel$, and hence of $\parallel \hat{{\boldsymbol{\theta }}}\parallel$.

#### RBFNN based sliding mode controller design

In order to take advantages of sliding mode control and RBFNN, we combine these two methods. That is, the nonlinear function *g*(*x*) is approximated by a RBFNN ${\hat{{\boldsymbol{\theta }}}}^{T}{\boldsymbol{\varphi }}(s)$ and the disturbances caused by *d*, ${\dot{y}}_{d}$ and approximation errors are dealt with by SMC −*ρ*sign(*s*). Then the actual control is designed as

23 $$\begin{array}{l}{u}_{a3}(t)=-\,\lambda s-{\hat{{\boldsymbol{\theta }}}}^{T}{\boldsymbol{\varphi }}(s)-\rho {\rm{sign}}(s)\end{array}$$

Considering the same Lyapunov function candidate as Eq.(16) and following the similar proof process, we can derive the stability condition

24 $$\begin{array}{l}|s| > ({k}_{c}{\theta }_{M}^{2}/4+{\omega }_{N}-\rho )/\lambda \end{array}$$

or

25 $$\begin{array}{l}\parallel \tilde{{\boldsymbol{\theta }}}\parallel  > {\theta }_{M}/2+\sqrt{({k}_{c}{\theta }_{M}^{2}/4+{\omega }_{N}-\rho )/{k}_{c}}\end{array}$$

Thus, if we choose $\rho  > {k}_{c}{\theta }_{M}^{2}/4+{\omega }_{N}$, the $\dot{V}\le 0$ can always be guaranteed and the tracking error will converge to zero.

In practice, it is important to avoid chattering phenomenon caused by discontinuous function −*ρ*sign(*s*). Here, we use a low-pass filtering (LPF) of the high-frequency switching term sign(*s*) in the control law Eq. (23). The LPF can be implemented as a first-order differential equation

26 $$\tau \dot{z}=-\,z+{\rm{sign}}(s)$$

where *τ* is a small positive scalar representing the time constant of the filter. Now, we obtain the final controller as

27 $$u=-\,\lambda s-{\hat{{\boldsymbol{\theta }}}}^{T}{\boldsymbol{\varphi }}(s)-\rho z$$

**Figure 8:**
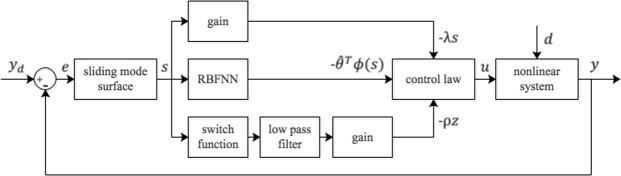
The control diagram constructed by feedback method, RBFNN and Sliding mode control. The random disturbance *d* is imposed to the RE and the control signals are applied on PY and IN. The tracking error is defined as *e* = *y* − *y*_*d*_ and taken as the sliding surface. And the control signal u includes three parts: the unknown nonlinear smooth function approximated by the RBFNN, the reaching law designed by feedback method and the filtered discontinuous function designed by SMC.

The overall control diagram is shown in Fig. 8.

### Control performance

To illustrate the effectiveness of the proposed control method on seizure abatement, we conduct several numerical experiments. In the simulations, the system under control is chosen as Eq. (1) with *c*_*tr*_ = 0.6 and *c*_*te*_ = 3 in bistable regimes with coexistence of SWD and background activity. Proper initial condition was chosen so that the system was in a SWD state. A Gaussian noise with mean 0 and standard deviation 1 and a randomly distributed pulses series were added to RE subpopulation to simulate background noises and unmodelled or unexpected projection from other brain areas respectively. In *Case 1*, the desired normal EEG signal *y*_*d*_ is generated using Eq. (1) with the same parameters *c*_*tr*_ = 0.6 and *c*_*te*_ = 3 but in a background state with properly chosen initial conditions. A Gaussian noise with mean 0 and standard deviation 1 is also added to RE subpopulation, mimicking background noises. In *Case 2*, we chose the desired EEG tracing as a channel clinical scalp EEG waveforms in alpha band from CHB-MIT database (https://www.physionet.org/pn6/chbmit/)^60^. Control parameters were chosen as *λ* = 1, *ρ* = 1, *k*_*c*_ = 1, *m* = 11, and *p* = 1 in all simulations.

**Figure 9:**
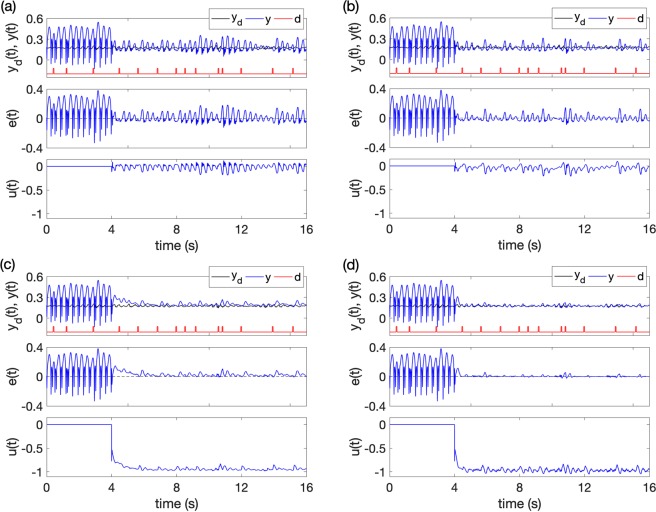
The control results. *Case 1*: the desired EEG tracing is in a background state. (**a)** With only feedback method, **(b)** with feedback and RBFNN methods, **(c)** with feedback and SMC methods, **(d)** with all the feedback control, RBFNN control and SMC methods. *y*_*d*_ is the expected normal output, *y* is actual output, *d* is the random pulses disturbance applied on RE. *e*(*t*) is the tracking error, *u*(*t*) is control signal. The control was switched on at *t* = 4s.

**Figure 10:**
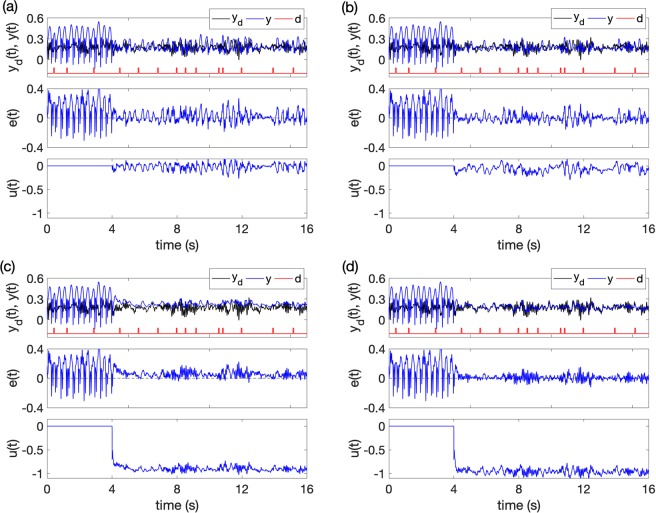
The control results. *Case 2*: the desired EEG tracing is in normal alpha band. **(a)** With only feedback method, **(b)** with feedback and RBFNN methods, **(c**) with feedback and SMC methods, **(d)** with all the feedback control, RBFNN control and SMC methods. *y*_*d*_ is the expected normal output, *y* is actual output, *d* is the random pulses disturbance applied on RE. *e*(*t*) is the tracking error, *u*(*t*) is control signal. The control was switched on at *t* = 4s.

Figures 9 and 10 give the control results of the two cases with different control methods: (a) method 1, with only the feedback control *u* = −*λs*, (b) method 2, with the feedback control and RBFNN *u* = −*λs* − *ρz*, (c) method 3, with the feedback control and SMC $u=-\,\lambda s-{\hat{{\boldsymbol{\theta }}}}^{T}{\boldsymbol{\varphi }}(s)$, and (d) method 4, with all the three controllers $u=-\,\lambda s-{\hat{{\boldsymbol{\theta }}}}^{T}{\boldsymbol{\varphi }}(s)-\rho z$. The controller was switched on at time *t* = 4s (The switch-on time has no effects on control performance, see Supplementary Fig. S2. As shown in Figs 9(a) and 10(a), in both *Case 1* and *Case 2*, the feedback control reduces the amplitudes of the pathological oscillations but can’t effectively suppress them. The effects of the random disturbances in RE can still evoke SWDs. When the feedback and RBFNN control methods are considered (see Figs 9(b) and 10(b)), the tracking performance is improved by RBFNN approximating the unmodeled dynamics, but the controlled system is still sensitive to external disturbances. The introduction of SMC, as shown in Figs 9(c) and 10(c), will further decrease the amplitudes of the oscillations and increase the robustness to external disturbances. But the tracking errors are still large. Finally, we combine all the three control methods to achieve best control performances, as shown in Figs 9(d) and 10(d), and the seizures are totally suppressed.

**Figure 11:**
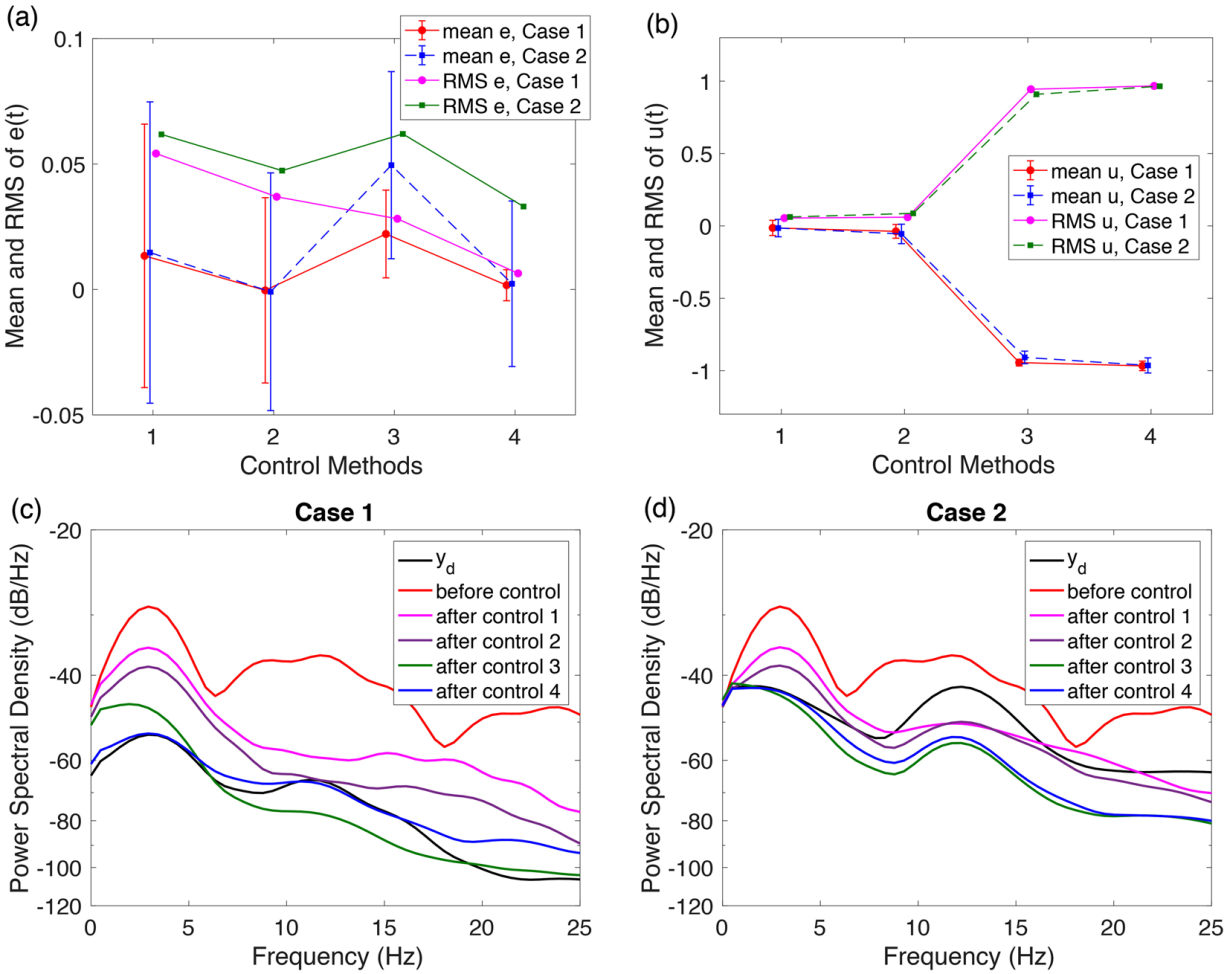
Control performance. (**a)** Root-mean-square (RMS) of tracking error (RMS e) and the mean and standard deviation of tracking error (mean e), **(b)** RMS of control *u* (RMS u) and the mean and standard deviation of *u*. These indexes are calculated using the last 12 seconds signals. **(c)** and **(d)** Power spectral density of different signals for *Case 1* and *Case 2* respectively. *Case 1*: the uncontrolled system is in a background state. *Case 2*: the desired EEG tracing is in normal alpha band. The four different control strategies are 1: feedback, 2: feedback and RBFNN, 3: feedback and SMC, and 4: feedback, RBFNN and SMC.

In order to intuitively represent the control performances of different control strategies, we calculated the root-mean-square (RMS) value and the mean value and standard deviation as performance indexes for the above two cases about the tracking error *e*(*t*) (RMS e and mean e in Fig. 11(a)) and the control signal *u*(*t*) (RMS u and mean u in Fig. 11(b)). The numbers in horizontal axis represent four different control methods (1: with only feedback, 2: with feedback and RBFNN, 3: with feedback and SMC, and 4: with feedback, RBFNN and SMC). All performances with different control methods keep consistent in *Case 1* and *Case 2*. As shown in Fig. 11(a), although method 2 has the lowest mean error, it has much bigger RMS e and standard deviation than method 4, which indicates its weaker robustness to external disturbances. Method 4 has very small mean errors, and the smallest RMS and standard deviation of *e*(*t*). The cost to get such a robust control result is the consumption of more control energy (see Fig. 11(b)). We also compared the power spectral density (PSD) of simulated EEG signals before and after control with that of the desired EEG as shown in Fig. 11(c,d) for *Case 1* and *Case 2* respectively. In *Case 1*, the desired EEG behaving as normal background activities shows no dominant frequencies and very small PSD (*dark line* in Fig. 11(c)), while in *Case 2* the desired EEG in alpha band shows dominant frequencies around 12 Hz as expected (*dark line* in Fig. 11(d)). Simulated abnormal EEG before control shows clear dominant frequencies around 3 Hz (*red lines*) as observed in clinical SWDs. Method 1 with only the feedback control (*purple lines*) and Method 2 with the addition of RBFNN suppresses the PSD but dominant frequencies around 3 Hz still exist (*brown lines*). The addition of SMC in method 3 further suppresses the PSD (*green lines*). Finally, the PSD of the signal after control by method 4 is similar to that of the desired normal EEG (*blue lines*). Considering all the indexes together, we conclude that method 4, taking advantages of feedback, RBFNN and SMC, provides the best control performance.

**Figure 12:**
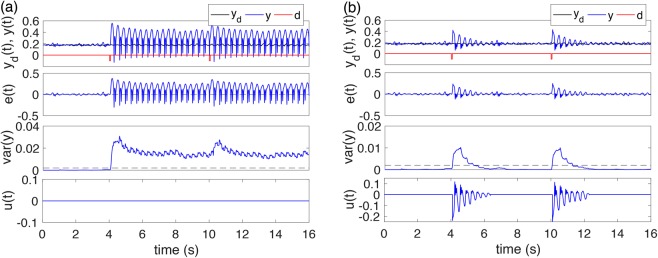
Variance-based on-demand closed-loop feedback control. (**a**) Without control. (**b**) With control.

The above simulations have demonstrated the effectiveness of our proposed control methods in seizure abatement even under big and frequent disturbances. The main advantage of this continuous closed-loop control is to guarantee seizure-free all the time. In practice, when the disturbance that causes seizures is not frequent, we may implement our control method in a responsive way and satisfactory seizure control may be obtained by using only the feedback control. That is, the control is only switched on when seizures are detected or predicted. As an attempt in this direction, we propose a variance-based on-demand closed-loop feedback control as shown in Fig. 12. We set the system in the bistability regime as that in Fig. 9. The desired EEG tracing *y*_*d*_(*t*) (*black lines*) is set as the background activity. We introduce the variance of simulated EEG *Var*(*y*) as a seizure indicator to determine the state of the system, where the variance is calculated using the moving window technique with window size 0.5 s and sliding step 0.05 s. When *Var*(*y*) > *Var*_th_ and lasts more than 0.1 s, seizures are believed to occur, and the feedback control is triggered on and continue in action for 1 s. As shown in Fig. 12(a), without control, initially the brain is in a state exhibiting normal background EEG activity (*t* < 4s), where the variance of simulated EEG *Var*(*y*) is very small, much lower than the threshold *Var*_th_ = 0.002. A single-pulse stimulation mimicking an unexpected disturbance may change the brain state from normal to SWD at *t* = 4s, whereafter the *Var*(*y*) is much bigger than the threshold *Var*_th_. A second single pulse stimulation at *t* = 10s) has little effects. Figure 12(b) shows the control results. Since the stability of control system is guaranteed, we can observe that with only feedback control for a short time period, the pathological activities can be well-controlled and the brain state is driven into the attraction basin of the background activity. Moreover, the control signal adaptively decays as the SWD is abated, which is suitable for energy-saving applications in clinical practice.

## Discussion

In this paper, we have investigated the complex states and various transitions in a thalamocortical computational model (Eq. (1)) of absence epilepsy by using bifurcation analysis. In order to understand the mechanisms of absence seizures including SWDs, many studies have been conducted on how the model parameters affect the states and their transitions of the system, such as the input constant parameter^61^, direct PY-to-TC excitation *c*_*te*_ and feedforward TC-to-PY inhibition *c*_*et*_^62^, and the feedforward inhibition and excitation from TC to cortex^63^. Motivated by the recent experimental results about the bidirectional communications of absence seizures in thalamocortical circuit^32,54,55^, we here focus on the top-down PY-to-TC excitation *c*_*te*_ and the feedforward RE-to-TC inhibition *c*_*tr*_. We have identified 10 qualitatively different regions in two-dimensional parameter space separated by Hopf bifurcation and double cycle bifurcation curves (Fig. 6). Various state transitions and dynamics including SWDs and tonic-clonic seizures and have been observed (Figs 2, 3, 5 and 6). The existence of multi-stable states (such as SWD, CO and LS in Fig. 7) are determined by the occurrence and relative positions of Hopf bifurcations and double cycles bifurcations, which are sensitive to parameter values. There are two-fold impacts of the characteristic multi-stability in absence epilepsy. On the one hand, it makes possible the use of single-pulse stimulations to successfully drive absence seizures to background activities^19,24^. On the other hand, there is no guarantee for the success of this kind of open-loop stimulation^24,64^. It is worth noting that the effect of single-pulse on eliminating pathological SWD is highly dependent on the initial states, parameter values and the imposed stimulation. For example, SWDs can be induced by single cortical pulses in a genetic absence animal model when the rats are in a drowsy state^64^. The previous proposed optimal single-pulse seizure abatement stimulations require the successful detection of the SWDs phase, which is difficult due to the complex dynamics of SWDs and ubiquitous noises^24,62^. We here also illustrate that the single-pulse stimulation may induce epileptic seizures instead of abating them (see Fig. 7), which indicates the unpredictable state transitions caused by open-loop direct stimulations. Therefore, closed-loop feedback control approach is necessary for reliable abatement of abnormal SW seizures.

Since the pioneering work of Schiff on neural control engineering^65,66^, several model-based control strategies have been proposed and tested on different neural systems^67,68^. In this paper, we have formulated the seizure abatement issue into a tracking control problem. We assume that there is a reference signal describing the normal background brain activities. The controller should be designed to drive the pathological activities to follow the desired normal activities. However, there are various uncertainties in neural systems, such as the dynamics of cell nonlinearity and residuals, the unmodeled dynamic characteristics, noises, and external disturbances^69^. At present, the stimulation used in the open-loop as well as the closed loop seizure control methods in animal experiments or clinical trials need to be pre-tested with trial-and-error to set good stimulation patterns and parameters, which remain unchanged during the treatment process^36–38,41–46^. Lack of adaptability and anti-interference ability makes seizure control with fixed stimulation prone to causing adverse events^38^. The previously proposed optimization control requires real-time detection of details about the epileptiform activity, such as the phase of seizure oscillations, which may not be accurately obtained due to the variability of seizure oscillations, noises and disturbances^24,49^. Consequently, we proposed RBFNN and SMC methods with feedback to construct the control strategy, where the RBF method can adaptively fit unmodeled dynamic characteristics^50,58,59^, and the SMC method has strong anti-interference ability^51,57^. Therefore, even for different patients or in different stages of seizure states, using the same control parameter settings, we can solve the tracking problem and achieve good seizure control results (see Figs 9–11 and Supplementary Figs S3–S5 for control of clonic seizures). The stabilities of the tracking error dynamics and the robustness of the control are guaranteed by Lyapunov stability theory, and thus the abatement for seizures can be obtained. As long as the control is on, seizures can always be suppressed and the states will maintain in the basin of the attractor of the desired normal activities. Our control design can be used to improve the robustness and adaptability of the current closed-loop seizure control systems^41–46^. Furthermore, the existing seizure detection algorithms^41–46,70,71^ can be easily implemented into our control system to further facilitate its practical applications (see Fig. 12). Put into other words, the proposed control method proves a universal and robust control strategy for brain stimulation of seizure abatement.

To implement the proposed seizure control methods in clinical practice, the desired EEG trace *y*_*d*_(*t*) should be firstly obtained. We suggest that one way is to pre-record a EEG signal of normal background activity of the patients. Another way is to online detect and classify the EEG signals into epileptic seizures and background activities, and choose one of the background activity traces as the reference signal *y*_*d*_(*t*). The control will be switched on for those brain areas with epileptic seizures. Optimal trade-offs between robustness and performance, which vary depending on patients’ conditions, should also be considered. SMC provides strong robustness and makes the closed-loop system insensitive to variations, while it also costs more energy (see Fig. 11). When big disturbances to the brain are rare, control methods without SMC implemented in a responsive way may also result in satisfactory seizure abatements with much less energy consumption (see Fig. 12(b)).

Several limitations in this study would be addressed in future research. First, there exist various models describing epileptic seizures at diverse physiological scales^10,11,72^. We have only examined the macroscopic behaviors from a nonlinear dynamics point of view. The model used in this paper can successfully reproduce resting background activity and paroxysmal activity like SWDs or COs, but no ongoing activity like alpha or beta waves in EEG. The results obtained by bifurcation analyses in this paper only provide qualitative but not quantitative reproduction or predictions of changes of brain states with varying connection parameters. Some of these predictions (like tri-stability regime) need further tests and observations in experimental or clinical studies. It will also be a big challenge to perform integrated qualitative and quantitative analyses of a multi-level computational model of epilepsy that reproduces various brain activities. Second, there are also many paths regulating the genesis and evolution of seizures in the cortico-thalamo-cortical loops^45,46,63,73–75^, and we have only investigated mechanisms of the top-town PY-to-TC excitation and feedforward RE-to-TC inhibition. The next step study would consider more comprehensive dynamical and physiological mechanisms of epileptic seizures with multiple changing parameters, and also other target brain regions for seizure control^45,46^. Third, we only consider one cortical column of brain area in this paper. Using spatially extended models with patient-derived connectivity^49,76–78^ and incorporating our method into the network control framework^79^ would be very promising. Last, we here mainly focus on the controller design for seizure abatement. On the other hand, seizures prediction and prevention has been a long challenge and it’s crucial to prevent the seizures before its clinical onset^80,81^. Recent analysis of EEGs have revealed that SWDs are preceded by precursor activity such as changes in delta-theta oscillations^71^ and synchrony^44,77^ in thalamocortical neural networks, which provides the feasibility of seizure prediction^81^. Implementation of advanced seizure prediction technologies^81^ into our control framework would be a significant step towards development of robust closed-loop seizure prevention systems.

## Supplementary information

Supplementary Information

## Data Availability

All data included in this publication or MatLab codes used for the analysis will be made available on reasonable request by contacting one of the corresponding authors.
