# Supplementary material for: Robust closed-loop control of spike-and-wave discharges in a thalamocortical computational model of absence epilepsy
Source: Sci Rep. 2019 Jun 24;9:9093. doi: 10.1038/s41598-019-45639-5 (PMC6591255; doi:10.1038/s41598-019-45639-5)
Supplement: Supplementary file 1 — Supplementary Information [file 41598_2019_45639_MOESM1_ESM.pdf]

Supplementary Information

Article in *Scientific Reports*

Robust closed-loop control of spike-and-wave discharges in a thalamocortical computational model of absence epilepsy

Yafang Ge, Yuzhen Cao, Guosheng Yi, Chunxiao Han, Yingmei Qin, Jiang Wang, Yanqiu Che

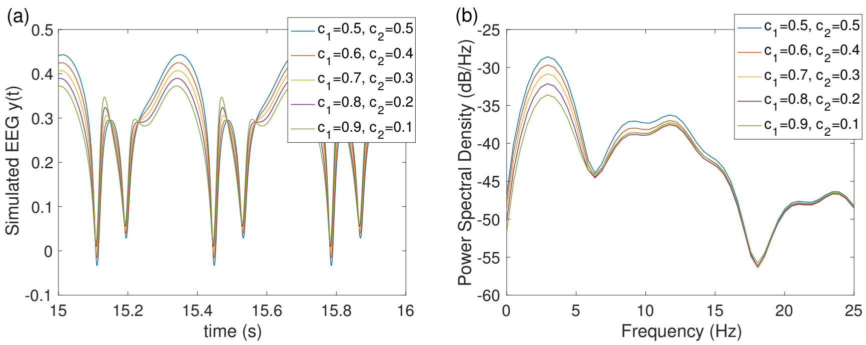

Supplementary Figure S1: **Effects of c_1, c_2 on the dynamics of simulated EEG $y(t) = c_1 \cdot PY + c_2 \cdot IN$.** **(a)** Waveforms of simulated EEG with different values of c_1 and c_2. **(b)** Frequency spectrum of simulated EEG with different values of c_1 and c_2. The changes of c_1 and c_2 have no qualitative effects on the dynamics of simulated EEG $y(t)$.

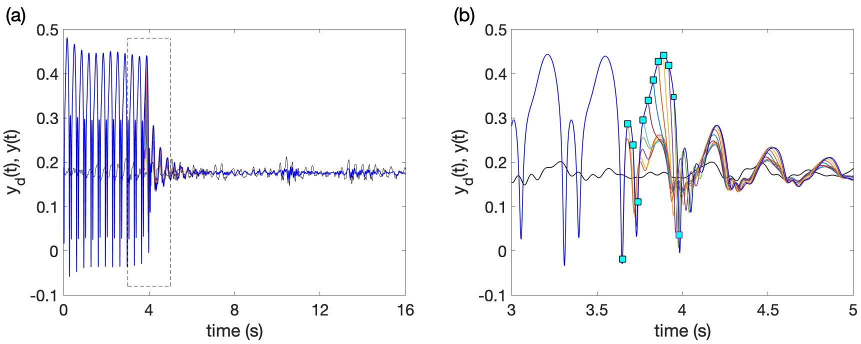

Supplementary Figure S2: **Robustness of the control system to control switch-on times.** **(a)** Waveforms of desired EEG y_d (*black line*) and EEG traces under control with different control switch-on times. **(b)** Enlargement of a part of (a) to show that the control is switched on at different phases of one period of SWD (*square box*). The control switch-on times has no effects on steady states and has very small effects on transient states.

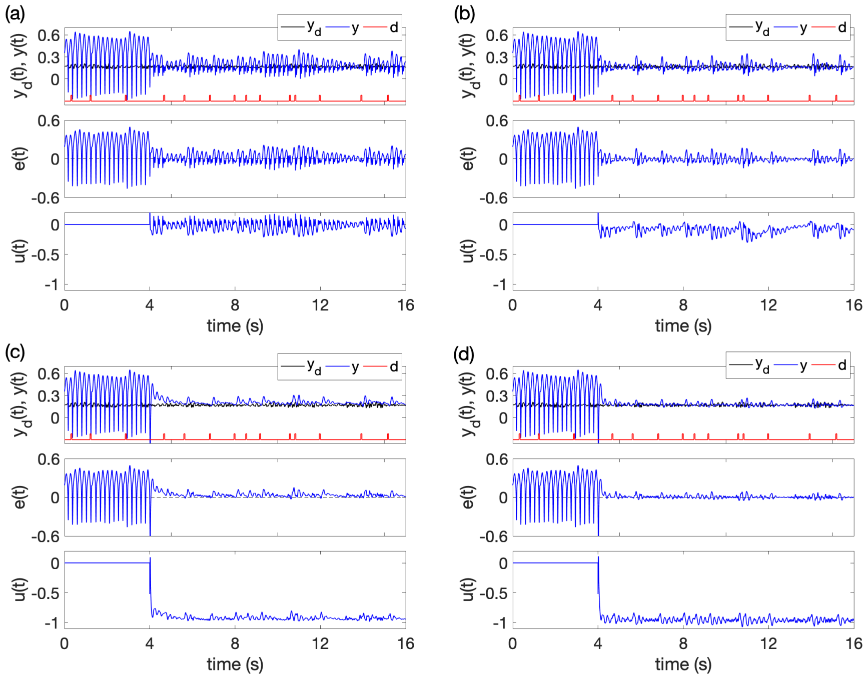

Supplementary Figure S3: **Abatement of clonic seizures.** ***Case 1: tracking normal background states*** **(a)** control method 1. **(b)** control method 2. **(c)** control method 3. **(d)** control method 4. The system under control is chosen as Eq. (1) with $c_{tr} = 1.5$ and $c_{te} = 3.5$ in tri-stable regimes with coexistence of SWD, clonic oscillations (CO) and background activity. Proper initial condition was chosen so that the system in a CO state.

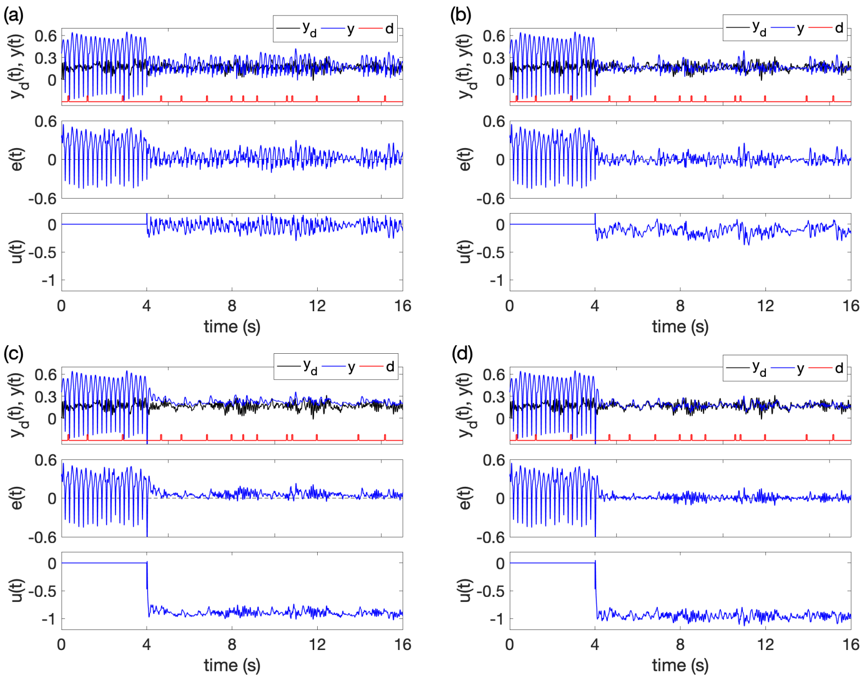

Supplementary Figure S4: **Abatement of clonic seizures.** ***Case 2:*** **tracking normal alpha EEG waveforms (a)** control method 1. **(b)** control method 2. **(c)** control method 3. **(d)** control method 4

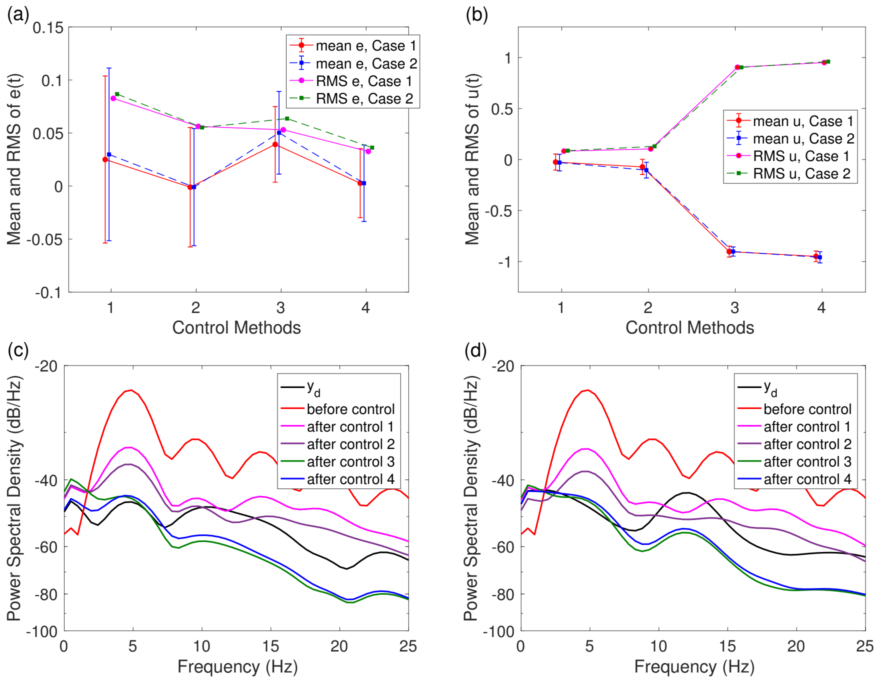

Supplementary Figure S5: **Abatement of clonic seizures. Control performance.** **(a)** Mean and RMS of $e(t)$. **(b)** Mean and RMS of $u(t)$. **(c)** Power spectrum density of $y_d(t)$ and EEG $y(t)$ before and after control for *Case 1*. **(d)** Power spectral density of $y_d(t)$ and EEG $y(t)$ before and after control for *Case 2*.
